# Genome Evolution

**DOI:** 10.1155/2010/643701

**Published:** 2011-07-21

**Authors:** Izabela Makałowska, Igor B. Rogozin, Wojciech Makałowski

**Affiliations:** ^1^Laboratory of Bioinformatics, Faculty of Biology, A. Mickiewicz University, 61-614 Poznań, Poland; ^2^National Center for Biotechnology Information, NIH, Bethesda, MD 20892, USA; ^3^Institute of Bioinformatics, University of Münster, 48149 Münster, Germany

Evolution is a central concept of biology; it explains both the diversity and the origin of all living organisms. It is based on the observation that change is a universal feature of nature. This idea is rooted in the philosophy of Heraclitus (535–475 BC) and is best expressed by the famous phrase, panta rei, coined by Simplicus in the sixth century AD. However, only modern biology has been able to explain how changes in biological systems occur. Genetic information is stored in long molecules of deoxyribonucleic acid (DNA). The complement of this information is called a genome and may consist of one or more DNA molecules, for instance, the human nuclear genome consists of twenty-three such molecules, called chromosomes. Interestingly, almost identical information in our closest relative (chimpanzees) is organized in twenty-four chromosomes. It is clear that, during evolution, genomes can undergo major rearrangements. These changes can be categorized into inversions, translocations, insertions, and deletions of genetic material that is piece of DNA. Many of these events are driven by repetitive sequences, most notably transposable elements. It should be noted, however, that minute changes at the DNA level, such as nucleotide substitutions and single nucleotide insertions or deletions, dominate the landscape of genomic changes. Nevertheless, it is fascinating to study all these changes and be able to infer the ancestral status of genomic content. 

With ever improving sequencing technology and the decline of the cost of sequencing, biologists are faced with a “data tsunami.” On the one hand, this constantly growing quantity of sequences and related information creates a real problem how to store and analyze such an amount of data. On the other hand, it gives us unprecedented opportunities to work on biological problems, which until recently were unsolvable. One such a problem, which is heavily data driven is genome evolution. At the moment of this writing (June 2011), there are over 4000 complete genome sequences listed in the NCBI's Entrez Genome: 2668 viral, 1656 microbial, and 42 eukaryotic (http://www.ncbi.nlm.nih.gov/genome/). Many more are under way, for instance over 700 eukaryotic genomes, including first genome scale population studies, 1000 human genomes project (http://www.1000genomes.org), and Drosophila Population Genomics Project (http://www.dpgp.org/). This indeed is an exciting time for those who study genome evolution. The last decade already witnessed enormous progress in understanding the structure and dynamics of genomes and with the current progress in molecular biology technologies we may expect another revolution in evolution research.

The presented special issue is dedicated to genome evolution and consists of six papers: one review, four research articles, and a resource review. The issue starts with a paper about the simplest organisms-viruses. C.-R. Huang and S. J. Lo discuss the evolution of the human hepatitis delta virus (HDV) genome, which, with a length of 1.7 kb, is the smallest known virus genome. HDV is not an autonomous virus since its genome does not code for the capsid protein, instead it uses an envelope protein of the hepatitis B virus (HBV) for its virion assembly. Hence, sometimes, it is called a satellite virus of HBV. Interestingly, HBV is the smallest know DNA virus with a genome spanning 3.2 kb. The authors explore a range of hypotheses on the HDV origin, evolution, and divergence.

Research papers in this issue of Advances in Bioinformatics cover a wide range of topics. C. S. M. Tang and R. J. Epstein searched the human genome for adaptive evolutionary hotspots and they found two separate ones that correlate with two extreme GC contents. Interestingly, these two extremes share many features, for example, intron length and gene expression level with genome isochores discovered by Bernardi in the 1970s. Based on the findings, they put forward a hypothesis about two mechanisms mediating adaptive evolution at the molecular level: “(1) intron lengthening and reduced repair in hypermethylated lowly-transcribed genes and (2) duplication and/or insertion events affecting highly-transcribed genes, creating low-essentiality satellite daughter genes in nearby regions of active chromatin.”

Annotating genes on newly sequenced genomes is one of the basic tasks in genome analysis. Yet, the current statistical methods fail to find complete sets of genes in a genome. J. Wu from the Carnegie Mellon University presents a new method to test protein coding potential of conserved short genomic sequences and applies it to the human genome. Adding conservation information to the statistical models of codons enables an increase of the number of candidate regions that can be coded for peptides and keeps the false positives rate relatively low. This new method was tested on the human genome with conservation information taken from human/mouse alignment. The procedure detected eighty-three percent of the human exons annotated in RefSeq collection, at a less than three percent false positive rate. J. Wu was able to determine 12,688 new short regions with protein-coding potential, most of which lay in the intergenic regions of the human genome. This is a promising observation since recent years witnessed a rapidly growing interest in long noncoding RNAs (lncRNAs), a relatively new actor on the genomic stage. However, despite many efforts, lncRNAs still hold a status of the genomic “dark matter.” Indeed, while other noncoding RNA molecules, that is, ribosomal, transfer, small nuclear, antisense, small nucleolar, micro-, and Piwi-interacting RNAs, have already been assigned well-defined functional roles, the origin and function of lncRNAs remain largely unknown. Even their definition is somewhat uncertain: lncRNAs are defined as noncoding transcripts longer than *∼*200 nucleotides. In addition, the evolutionary conservation of many lncRNAs is poor, they do not appear to be under direct selection, and the levels of their expression are low. It cannot be excluded that at least some lncRNAs encode unknown short proteins, thus prediction of protein-coding regions is still an important avenue of research.

The fast growing field of evolutionary medicine is promising a better understanding of infectious diseases. After all, medicine is based on biology and the two fields can only be fully integrated within an evolutionary framework. Developing a new vaccine is not a trivial task. Some fast evolving pathogens, for example, HIV, notoriously escape our efforts to develop an effective approach. M. S. Abu-Asab et al. explore a selective pressure induced by a vaccine on infecting bacterial strains and its implication on vaccine design. They developed a phylogenetic approach to understand why a vaccine had not worked. They used predicted pilin sequences on a phylogenetic tree to assess the vaccine's effect on Neisseria strains, in particular if the vaccine has caused an increased selection pressure on the pathogen. This method should help to reformulate vaccine design for the next round of trials. This paper clearly shows the importance of basic science in any applied field and medicine in particular. 

One of the first tasks after obtaining sequences is to assemble them into longer pieces with the ultimate goal to obtain a complete genome. However, nowadays, when a whole shotgun strategy dominates, the order of the sequenced pieces is unknown, making assembly challenging. The usual strategy is to assemble sequences based on sequence overlaps and clone-size information. In a new approach, M. Peto et al. explore the usefulness of DNA signatures, defined as distribution of dinucleotides, in assembly of chromosome sequences. This method aims at overcoming difficulties in the assembly of genomic sequences in the centromeric and pericentromeric regions caused by a lack of recombination events in these areas. The authors used dinucleotide signature and binding energy to aid soybean genome assembly. This interesting method should be especially useful in the detection of misassembly and may be further improved by the incorporation of other genomic signals, for example nucleosome binding potential.

This issue is concluded by a paper by L. Carmel and colleagues that discusses EREM software that uses maximum likelihood to estimate the parameters of a probabilistic model of binary character evolution on a bifurcating phylogenetic tree. This program was successfully applied to sets of conserved genes from nineteen eukaryotic species. It was inferred that a relatively high intron density was reached early; that is, the last common ancestor of eukaryotes contained more than 2.2 introns per kilobase, a greater intron density than in many extant fungi and some animals. The rates of intron gain and intron loss appear to have been dropping during approximately the last one billion years, with the decline in the gain rate being much steeper. It seems that intron gain has been episodic and, perhaps, associated with major evolutionary transitions, for example, the origin of animals, as opposed to the more uniform (even if lineage specific) intron loss process. Indeed, it appears certain that, for example, during the evolution of mammals (*∼*100 million years) and, probably, during the evolution of vertebrates (over 400 million years), there has been virtually no intron gain. Other eukaryotic lineages might have a higher intron gain rate, though, as illustrated by the evidence of apparent recent gain in nematodes. In addition to the analysis of introns, EREM can be applied to various binary characters, for example, gene content and morphological characters.

It is worth noting that all the papers presented here were written over a year ago at the dawn of new sequencing methods, which no doubt will bring new computational challenges that will need to be addressed by the genomic community to successfully utilize the accumulated data. However, we have no doubt that the approaches described in this special issue will be widely used by the scientific community in the near future. 

*Izabela Makałowska* *Igor B. Rogozin* *Wojciech Makałowski*

